# The Relationships between Plant Developmental Traits and Winter Field Survival in Rye (*Secale cereale* L.)

**DOI:** 10.3390/plants10112455

**Published:** 2021-11-13

**Authors:** Hirbod Bahrani, Monica Båga, Jamie Larsen, Robert J. Graf, Andre Laroche, Ravindra N. Chibbar

**Affiliations:** 1 Department of Plant Sciences, University of Saskatchewan, Saskatoon, SK S7N 5A8, Canada; hib860@mail.usask.ca (H.B.); monica.baga@usask.ca (M.B.); 2 Harrow Research and Development Centre, Agriculture and Agri-Food Canada, Harrow, ON N0R 1G0, Canada; jamie.larsen@canada.ca; 3 Lethbridge Research and Development Centre, Agriculture and Agri-Food Canada, Lethbridge, AB T1J 4B1, Canada; robert.graf@canada.ca (R.J.G.); andre.laroche@canada.ca (A.L.)

**Keywords:** winter cereals, winter field survival, low temperature tolerance, final leaf number, plant height, prostrate growth habit

## Abstract

Overwintering cereals accumulate low temperature tolerance (LTT) during cold acclimation in the autumn. Simultaneously, the plants adjust to the colder season by making developmental changes at the shoot apical meristem. These processes lead to higher winter hardiness in winter rye varieties (*Secale cereale* L.) adapted to Northern latitudes as compared to other cereal crops. To dissect the winter-hardiness trait in rye, a panel of 96 genotypes of different origins and growth habits was assessed for winter field survival (WFS), LTT, and six developmental traits. Best Linear Unbiased Estimates for WFS determined from five field trials correlated strongly with LTT (r = 0.90, *p* < 0.001); thus, cold acclimation efficiency was the major contributor to WFS. WFS also correlated strongly (*p* < 0.001) with final leaf number (r = 0.80), prostrate growth habit (r = 0.61), plant height (r = 0.34), but showed weaker associations with top internode length (r = 0.30, *p <* 0.01) and days to anthesis (r = 0.25, *p <* 0.05). The heritability estimates (*h^2^*) for WFS-associated traits ranged from 0.45 (prostrate growth habit) to 0.81 (final leaf number) and were overall higher than for WFS (*h*^2^ = 0.48). All developmental traits associated with WFS and LTT are postulated to be regulated by phytohormone levels at shoot apical meristem.

## 1. Introduction

Rye (*Secale cereale* L.) is an annual grass of the *Triticeae* tribe within the *Pooideae* subfamily, which also includes barley (*Hordeum vulgare* L.), hexaploid wheat (*Triticum aestivum* L.), and several pasture grasses. The cultivated form of rye (*Secale cereale* ssp. *cereal*) is mainly grown in North America, Northern and Eastern Europe, Russia, and China, where the grain is used for animal feed or production of bread and alcoholic beverages. Rye can also be grown for biomass used as forage, green manure, or production of bioenergy [1,2]. Cultivation of rye is traditionally performed with open-pollinated breeding populations, but they are gradually being replaced by hybrid varieties producing higher yields due to heterosis effects [3]. Rye has a relatively high drought tolerance due to a well-developed root system and is therefore often cultivated on marginal land that is unsuitable for most other cereal crops [4]. The undemanding nature of rye is an important asset for future development of new varieties that will withstand the effects of climate change.

The growth habit of annual temperate cereals can broadly be divided into winter, facultative, and spring types, which differ in seeding time due to variation in vernalization and photoperiod sensitivities. The most commonly grown rye varieties are autumn-seeded, frost-tolerant winter types, which have a vernalization requirement prior to winter and flower when long days and warmer temperature return in the spring. Facultative types, sown in the spring or autumn, are photoperiod sensitive, lack vernalization requirement, but can develop low temperature tolerance (LTT) by cold acclimation [5]. Spring-seeded genotypes lack or have very low vernalization requirement, and thus are established and set seed in a single growing season. In contrast to annual rye, vernalization-dependent perennial rye (*Secale cereale* × *Secale strictum*) re-enters a vegetative state after seed set, which is a growth habit dependent on both environmental and genetic factors [6].

Vernalization in *Triticeae* winter types is required for flowering competency and it is induced by low, nonfreezing temperatures in the autumn. The process is largely controlled by the *VERNALIZATION 1* (*VRN1*) and *VRN2* genes that are part of the *VRN1*-*VRN2*-*VRN3* regulatory module [7,8,9,10,11]. Cold exposure leads to vernalization saturation characterized by induction of *VRN1* expression at shoot apical meristem (SAM) and leaves, whereas *VRN2*, the main repressor of flowering, becomes down-regulated [9,12,13]. At vernalization saturation in the late autumn, SAM switches from vegetative to reproductive growth [14] and *VRN1* and *VRN3* are imprinted with an epigenetic ‘memory of cold’ that is maintained throughout vegetative plant growth [15]. The identification of Arabidopsis *FLOWERING LOCUS C* (*FLC*)-like genes in monocots with demonstrated roles during vernalization [8,16,17,18] has complicated the original *VRN* regulatory model and a full understanding of the vernalization process has not been obtained [19].

The low temperature-induced cold acclimation is a process genetically linked to vernalization [20,21]. Many cryoprotective compounds are produced during cold acclimation, which readjust metabolism, photosynthesis, and membrane composition to build up LTT prior to winter [22,23,24]. A cluster of *C-REPEAT BINDING FACTOR* (*CBF*) genes at *Frost Resistance 2* (*FR-2*) locus on homoeologous group 5 chromosomes in *Triticeae* species induce many cold-responsive (*COR*) genes during cold acclimation [25,26,27]. Genetic factors influencing vernalization sensitivity, photoperiod (day length) response, and cold acclimation efficiency affect winter field survival (WFS) potential in cereals [28].

Rye is a good model crop to study WFS as certain rye varieties adapted to northern latitudes exhibit the strongest vernalization requirement, highest cold acclimation efficiency, and highest WFS among cereals [29]. Determination of WFS levels in cereals is generally achieved by multi-year trials, which are time-consuming and subject to large year-to-year variations due to environmental factors. However, an approximation of LTT developed prior to winter can be obtained by controlled freezing tests performed on cold acclimated crown tissues [14] or plantlets [30]. The accumulated LTT in late autumn gradually declines during winter at a rate determined by the plants de-acclimation resilience and environmental factors such as the amount of snow cover, number of freeze–thaw incidents, and freezing temperatures at crown level [31]. Snow mold infections can also cause LTT decline, thus drastically reduce WFS in locations with long-lasting wet cool weather and deep snow coverage [32]. Production of winter cereals is expected to become more challenging in the future as the winter temperatures become more variable and unpredictable due to global warming [33,34]. Thus, de-acclimation resistance and ability to combat fungal infections will become important components of WFS for winter cereals [31,32].

In winter wheat, the cold acclimation process is highly integrated with the developmental program of the plant [35,36,37]. This is demonstrated by many genotypes with high vernalization requirement exhibiting a long cold acclimation process, whereby a prostrate growth habit (PGH) is developed and a high number of leaf primordia are initiated at SAM, which lead to high final leaf number (FLN) at maturity [14,35,37]. The PGH displayed prior to winter is an adaptive response proposed to suppress weed growth, reduce water evaporation from soil, increase photosynthetic efficiency, and allow plants to benefit from warmer temperature and reduced wind exposure at ground level [38,39]. During reproductive growth in the spring, the winter-hardy cereals have tendency to flower late, grow tall and produce small and narrow leaves [37].

The flag leaf provides most of the photosynthate required for grain filling in wheat cultivars [40]. However, the rye flag leaf area (FLA) is relatively small and occupies only 15–20% of the total photosynthetic area of the plant [4]. As most open-pollinated rye varieties are tall (about 120–150 cm), the photosynthetic area of stem becomes highly significant (60–80% of total) and stem is therefore considered the main producer of photosynthate for grain filling [4]. The top internode (peduncle) elongates last during stem growth and has a major influence on the final plant height (PHT) [41,42]. Tall plants are generally desired for biomass production [1], but less advantageous for grain production as long peduncles can cause lodging with negative effects on grain quality and yield. Reduction in plant height in rye by introgression of dwarfing genes, such as *Ddw1* [43], have not resulted in similar yield increases obtained by semi-dwarf wheat [44], which commonly carries various height-reducing alleles [45,46]. Observations in winter wheat support semi-dwarfing genes do not negatively influence LTT [37]; thus, the search for new sources of height-reducing genes for winter rye continues.

In this study, a rye panel of 96 genotypes of diverse geographic regions, growth habits, and winter hardiness levels was analyzed for WFS during five years of field trials. The genotypes were also assessed for LTT (LT_50_ values) and six developmental traits to characterize their association with WFS. The results showed that LTT contributed most to high WFS, and several of the plant developmental traits showed very strong association with WFS and LTT.

## 2. Results

### 2.1. A Multi-Trait Approach to Study WFS

In this study, WFS in a rye population of 96 rye genotypes (Table 1) was studied during five years of field tests (Figure S1). To dissect the main trait further, we assessed traits associated with WFS, which is a strategy that can be very informative when analyzing complex traits [47]. Therefore, the WFS studies were accompanied by determinations of LTT, FLN, PGH, days to anthesis (DTA), and PHT, which associate with winter hardiness in certain winter wheat genotypes [14,35,37]. Additionally, top internode length (TIL) and flag leaf area (FLA), which are not commonly related to WFS, were also analyzed. Phenotyping of LTT and the developmental traits is relatively easy to accomplish when compared to WFS, which can vary largely from year to year due to abiotic and biotic stress factors affecting plant survival from the early seedling stage in autumn to spring regrowth.

### 2.2. WFS for Rye Population Displayed Large Variations between Years

During the five-year field trials performed at Saskatoon, Canada, the snow cover and winter temperatures were highly variable between years (Table S1). The highest WFS was obtained during the 2015/2016 growing season (92.6% average WFS; Figure S1), which provided adequate snow cover and a relatively mild winter.

**Table 1:** Rye genotypes used in the study and their WFS and LT_50_ scores.

Winter Survival Class: Genotype	Origin	Growth Habit	WFS BLUE Score *	LT_50_ Value (°C)
**Very high:**				
Leth Coulee Rye	Canada	Winter	92.5	−26.8
Gauthier	Canada	Winter	90.1	−26.2
AC Remington	Canada	Winter	86.2	−27.0
AC Rifle	Canada	Winter	85.9	−27.0
Musketeer	Canada	Winter	83.0	−27.8
SM 38R	Canada	Winter	77.5	−24.0
Prima	Canada	Winter	77.0	−27.5
Saratovskaja 4	Russia	Winter	71.8	−26.8
SM 4R	Canada	Winter	71.0	−26.8
Pearl	Denmark	Winter	69.5	−26.6
Kustro	Canada	Winter	68.8	−25.8
Kharkivska 95	Ukraine	Winter	67.9	−24.8
Kharkivska 98	Ukraine	Winter	66.9	−24.0
Esprit	Germany	Winter	66.3	−22.8
Ponsi	Sweden	Winter	66.0	−24.8
Hazlet	Canada	Winter	65.5	−23.6
Antelope	Canada	Winter	65.3	−26.2
Emerald	USA	Winter	65.2	−22.0
Anna	Finland	Winter	64.5	−22.0
**High:**				
R003-4	Canada	Winter	64.3	−24.0
Voima	Finland	Winter	64.2	−23.8
Dakota	Canada	Winter	64.1	−26.7
Sc-73	Canada	Winter	64.0	−22.4
Animo	Netherlands	Winter	63.6	−25.2
Caribou	Canada	Winter	63.6	−23.8
Puma	Canada	Winter	62.4	−26.0
Othello	Sweden	Winter	62.2	−22.0
Rymin	USA	Winter	61.9	−23.4
Adams	USA	Winter	61.5	−22.8
Sangaste	Estonia	Winter	60.3	−23.0
Visa	Finland	Winter	59.9	−24.2
Vitallo	Germany	Winter	59.6	−23.5
Halo	Germany	Winter	59.5	−26.2
Balbo	Italy	Facultative	59.4	−26.0
Frontier	Canada	Winter	58.6	−24.4
Enzi	Finland	Winter	58.4	−22.0
Explorer	USA	Facultative	58.4	−23.4
Motto	Poland	Winter	58.0	−23.6
Dankowskie Selekcyjne	Poland	Winter	56.7	−23.8
**Moderate:**				
Galma	Belgium	Winter	56.6	−22.6
Cougar	Canada	Winter	56.1	−24.0
Dominant	Netherlands	Winter	55.8	−23.6
Dankowskie Nowe	Poland	Winter	54.9	−24.6
Danko	Canada	Winter	54.2	−24.8
ACE-1	Canada	Perennial	54.0	−19.4
Dankowskie Srebrne	Poland	Winter	53.9	−24.2
Carolkurz	Germany	Winter	53.2	−23.8
Horton	Canada	Winter	53.1	−24.0
Kodiak	Canada	Winter	51.8	−25.0
GC-100	Russia	Winter	51.6	−23.0
Amilo	Poland	Winter	49.2	−20.8
Sellino	Germany	Winter	48.5	−21.8
R538	UK	Perennial	48.1	−21.6
Protector	Germany	Winter	47.8	−22.4
Toivo	Finland	Winter	47.5	−23.8
Culpan	Russia	Winter	47.0	−22.6
Hardy white spring Rye	Austria	Winter	46.9	−21.6
Maton	USA	Facultative	46.2	−19.5
**Low:**				
Stoir	Ukraine	Winter	43.7	−22.2
Vaschod	Belarus	Winter	43.7	−21.2
R550	Czech Republic	Perennial	43.6	−21.4
Reimann Philipp	Germany	Perennial	42.4	−21.0
Oklon	USA	Facultative	40.9	−19.6
Carsten	Germany	Winter	39.5	−18.0
R903	Unknown	Perennial	38.9	−22.0
Harach	Canada	Spring	38.8	−21.4
Danae	Germany	Winter	37.1	−21.2
Clse 35	USA	Winter	36.8	−20.2
Gator	USA	Facultative	36.0	−23.2
Elbon	USA	Facultative	35.9	−17.0
L-286-R	Germany	Winter	35.7	−16.4
R904	Unknown	Perennial	35.4	−19.8
Syn 20-L	Germany	Winter	35.3	−21.8
SR4A-S5	Canada	Spring	33.2	−17.6
Dakold	USA	Winter	31.1	−20.5
Wheeler	USA	Winter	31.0	−20.8
M.Karlic CT2	Russia	Winter	30.5	−19.5
**Very low:**				
Wintergrazer 70	USA	Facultative	25.2	−20.2
Petkus Kurzstroh	Germany	Winter	24.1	−19.0
Gazelle	Canada	Spring	23.6	−19.0
Petkus	Germany	Winter	22.9	−21.2
Prolific Spring	Canada	Spring	22.1	−19.2
Wren Abruzzi	USA	Facultative	20.0	−18.0
Extra Early Rye1	Mexico	Spring	19.7	−16.4
Somro	Germany	Winter	16.0	−18.8
R1210	South Africa	Perennial	15.7	−16.0
Baltia	Russia	Winter	15.6	−16.8
R797	Poland	Perennial	13.2	−16.0
Fl-Synt	USA	Spring	12.9	−16.4
Ottawa Select	Canada	Winter	12.9	−16.8
Gulzow Kunz CT1	Germany	Winter	12.4	−16.2
Rogo	Germany	Spring	12.4	−16.2
Florida 401	USA	Spring	7.1	−15.8
L-145-N	Germany	Winter	0.0	−17.0
L-145-P	Germany	Winter	0.0	−16.5
L-18-R	Germany	Winter	0.0	−16.5

* WFS data from five field trials; includes previous data from four trials [48].

In contrast, survival upon the 2017/2018 winter season was very low (6.8% average WFS) due to deeper and longer cold spells and occasional poor snow cover. The remaining trials in 2014/15, 2016/17, and 2018/19 provide a desired wide distribution of WFS values within the population. Despite the challenges with WFS phenotyping in certain years, most of the WFS data from the trials were significantly correlated (Table 2). The 2016/2017 and 2018/2019 trials showed the strongest correlation (r = 0.67, *p* < 0.001), whereas the 2015/2016 and 2017/2018 trials did not significantly correlate with each other (r = 0.17, *p* > 0.05).

**Figure 1:**
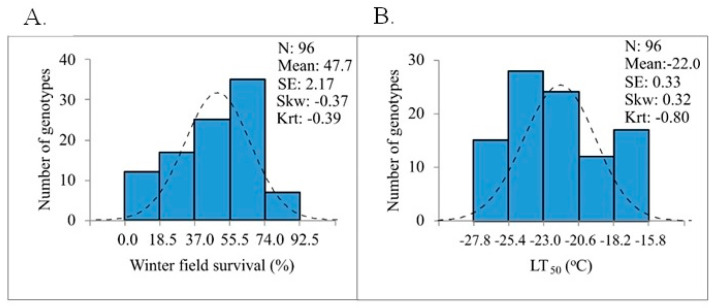
Distribution of frost hardiness determined for rye population of 96 accessions. (**A**) WFS (BLUE scores) determined from five years of field trials; (**B**) average LT_50_ values determined from controlled freezing tests.

The BLUEs calculated for WFS from all five trials ranged from 0.0 to 92.5% (Table 1) with a mean of 47.7% (Figure 1A). The correlation value between WFS_BLUEs and the WFS values for the five individual trials were highly significant (*p* < 0.001) and ranged from 0.59 to 0.91 and were overall higher than observed between the individual trials (Table 2). Winter types, as expected, showed the highest WFS (WFS_BLUE = 0.0–92.5%; mean = 52.8%), followed by facultative types (20.0–59.4%; mean = 40.3%), perennial types (13.2–54.0%; mean = 36.4%), and spring types (7.1–38.8%; mean = 21.2%) (Table 1). From the WFS_BLUEs, five WFS classes were defined for the rye panel: (i) very high (64.5–92.5%; 19 genotypes), (ii) high (56.7–64.3%; 20 genotypes), (iii) moderate (46.2–56.6%; 19 genotypes), (iv) low (30.5–43.7%; 19 genotypes), and (v) very low (0.0–25.2%; 19 genotypes) (Table 1). Eleven of the nineteen genotypes within the very high WFS class were winter types adapted to the cold Canadian winters (Leth Coulee Rye, Gauthier, AC Remington, AC Rifle, Musketeer, SM 38R, Prima, SM 4R, Kustro, Hazlet, and Antilope). Carsten and Petkus, which have frequently been used for cultivation and breeding in Europe [4], were placed in the low and very low WFS classes, respectively (Table 1).

### 2.3. Freezing Tests Provided a Good Estimate of WFS Levels for the Rye Genotypes

One component of WFS is LTT built up during cold acclimation prior to winter and this trait was estimated by controlled freezing tests. Cold-hardy wheat cultivar Norstar used as a control in the tests showed an average LT_50_ value of −21.4 °C, whereas higher LTT (lower LT_50_ values) was noted for 59 out of 96 (61%) rye genotypes tested (Table 1). Genotypes of the very high WFS class had the highest LTT (LT_50_mean_ = −25.4 °C) and a gradual decrease in average LTT was seen for the following WFS classes: high WFS (LT_50__mean = −24.0 °C), moderate WFS (LT_50__mean = −22.8 °C), low WFS (LT_50__mean = −20.3 °C) and very low WFS (LT_50__mean = −17.5 °C) (Table 1). Like the WFS distribution, the absolute skewness and kurtosis values for LT_50_ distribution were <1.0, which was indicative of continuous trait variation within the population (Figure 1A,B).

The freezing test data for the rye population confirmed winter types developed the lowest LT_50_ values (e.g., highest LTT) during cold acclimation (Table 1) and highest LTT was noted among the most winter-hardy genotypes such as Canadian cultivars Musketeer (LT_50_ = −27.8 °C), Prima (LT_50_ = −27.5 °C), AC Remington (LT_50_ = −27.0 °C), and AC Rifle (LT_50_ = −27.0 °C). However, a few winter types such as Baltia, Ottawa Select, Gulzow Kunz CT1, and three highly inbred lines (94-L-145-N, 95-L-145-P, and 96-L-18-R) exhibited very low LTT (LT_50_ > −17.0 °C) and also survived poorly in the field (WFS_BLUE < 15.6%) (Table 1). The facultative genotypes in the study showed overall low WFS with the exception of the genotypes Balbo (WFS_BLUE = 59.4%; LT_50_ = −26.0 °C) and Explorer (WFS_BLUE = 58.4%; LT_50_ = −23.4 °C), which were included in the high WFS class (Table 1). Among the perennial genotypes, the Canadian ACE-1 developed for pasture and silage production [49] performed best, but did not accumulate adequate winter hardiness prior to winter to survive well on the Canadian Prairies (WFS_BLUE = 54.0%; LT_50_ = −19.4 °C; Table 1). As expected, the lowest WFS and LTT were recorded for spring genotypes (WFS_BLUE ≤ 38.8%; LT_50_ ≥ −21.4 °C; Table 1), for which seven out of eight genotypes did not survive the 2017/18 winter (data not shown). Overall, LTT determined for the rye population showed very high correlation with WFS_BLUEs (r = 0.90; *p* < 0.001; Table 3).

### 2.4. FLN and PGH Values Were Strongly Associated with WFS

**Figure 2:**
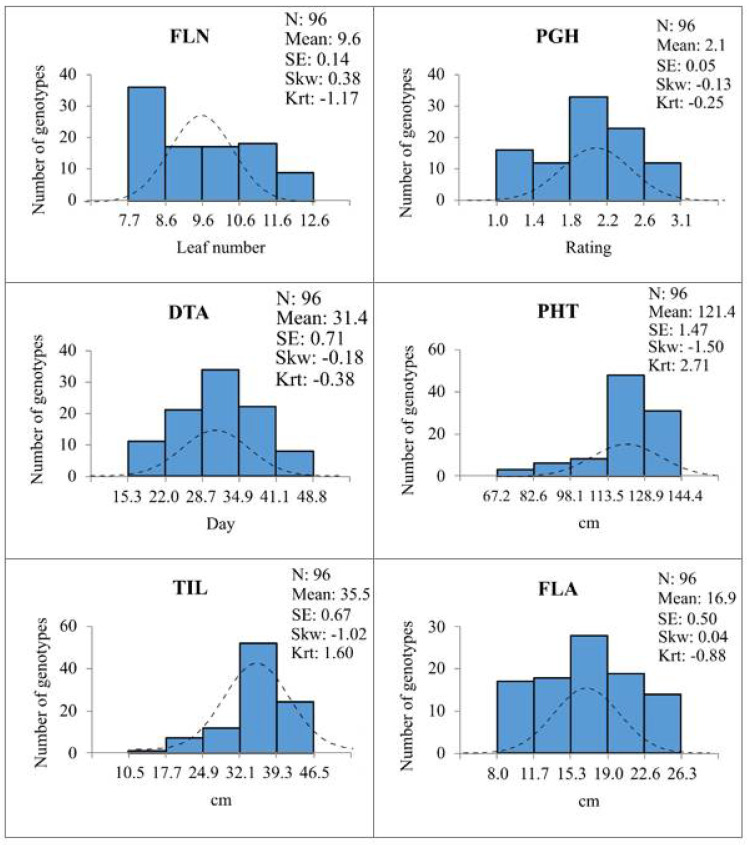
Distribution of developmental trait values for rye population of 96 accessions. Histograms show BLUE scores calculated for final leaf number (FLN), prostrate growth habit (PGH), days to anthesis (DTA), plant height (PHT), top internode length (TIL), and flag leaf area (FLA) determined from four independent trials.

Among the rye genotypes FLN_BLUEs varied from 7.7 to 12.4 leaves per plant with a mean 9.6 leaves (Figure 2 and Figure S2; Table S2). High FLN (>11 leaves) was observed for 11 genotypes of the very high WFS class (19 genotypes), whereas all genotypes within the very low WFS class (19 genotypes) produced less than nine leaves (Table S2). Perennial, facultative and spring genotypes had overall lower FLN than winter types (Table S2). Rye genotypes Musketeer, AC Rifle, AC Remington with the highest FLN (≥12.0 leaves; Table S2) were among the most winter-hardy (WFS ≥ 83%) and developed highest LTT during cold acclimation (LT_50_ < −27.0 °C) (Table 1). In contrast, breeding lines Petkus and Carsten had relatively low FLN (8.0 and 8.6, respectively; Table S2) combined with low winter-hardiness and relatively high LT_50_ values (Table 1). The FLN values determined for the rye population were strongly associated (*p* < 0.001) with WFS (r = 0.80) and LTT (r = 0.71) values, respectively (Table 3).

For individual plants, PGH was determined by visual scoring of cold-acclimated plants for their growth habit according to a scale ranging from erect (1), intermediate (2), or clearly prostrate (3) (Figure S3). Data from the four trials generated PGH_BLUE values ranging from 1.0 to 3.1 with an average of 2.1 for the rye population (Figure 2 and Figure S3). The winter and perennial genotypes showed overall higher PGH scores (mean 2.1) than the facultative and spring types (mean ~1.6; Table S2). PGH showed strong correlations (*p* < 0.001) with WFS (r = 0.61), LTT (r = 0.59), FLN (r = 0.43), and DTA (r = 0.43) (Table 3).

### 2.5. Delayed Anthesis Time Was Weakly Associated with Higher WFS

The plants that had undergone vernalization under controlled conditions were used to determine DTA, by counting the days from the end of cold acclimation to the start of anthesis. Values ranging from 15.3 to 48.8 days with a mean of 31.4 days were obtained for the rye population (Figure 2 and Figure S4; Table S2). With the exception of genotype SR4A-S5, the spring lines flowered early (DTA <26 days) and the longest delay to flowering was noted for the perennial genotypes, which all needed at least 34 days to reach anthesis (Table S2). DTA showed relatively weak association with WFS (r = 0.25, *p* < 0.05), but no significant association with LTT, and was negatively associated with both PHT (r = −0.29, *p* < 0.01) and TIL (r = −0.25, *p* < 0.05) (Table 3). However, a relatively strong association (*p* < 0.001) was noted between DTA and PGH (r = 0.43; Table 3).

### 2.6. Higher WFS Was Associated with Genotypes Growing Tall

Data from four trials were used to calculate PHT_BLUEs and TIL-BLUEs (Figure S5 and S6) and were found to be highly correlated (*p* = 0.001; r = 0.72; Table 3). The PHT_BLUEs varied from 62.7 to 144.4 cm with a mean of 121.4 cm and variation for TIL_BLUEs ranged from 10.5 to 46.5 cm with a mean of 35.5 cm (Figure 2; Table S2). TIL values constituted 15.6 to 38.3 % of the total height for the rye genotypes. Genotypes of short stature (PHT < 100 cm) were only identified among the spring and winter types (Table S2). Both PHT and TIL showed similar associations with WFS (r = 0.34, *p* < 0.001 versus 0.30, *p* < 0.01) and LTT (r = 0.39 versus 0.36, *p* < 0.001) (Table 3).

### 2.7. Variation for FLA Did Not Relate to Winter-Hardiness

Field grown plants from four trials were used to determine FLA_BLUEs (Figure S7) and showed a normal distribution with areas varying from 8.0 to 26.3 cm^2^ and a mean of 16.9 cm^2^ (Figure 2; Table S2). FLA showed no significant (*p* < 0.05) association with any of the other traits studied (Table 3), and thus did not seem to be affected by factors controlling cold acclimation.

### 2.8. Bi-Plot PCA Supported WFS Is Primarily Determined by Developments at SAM during Cold Acclimation

**Figure 3:**
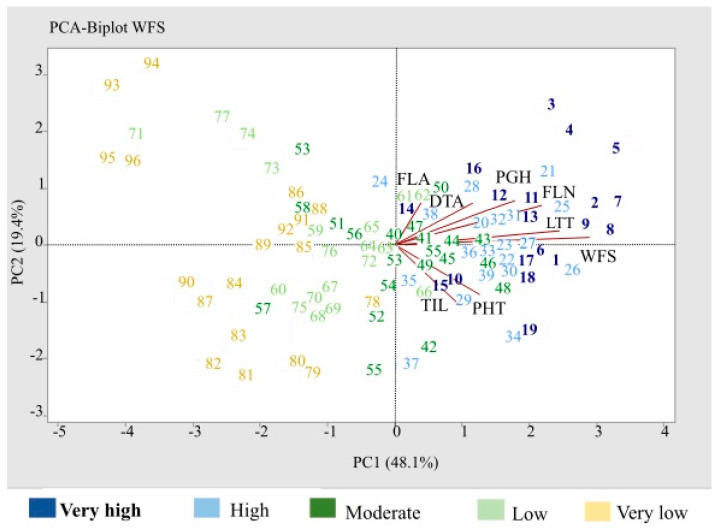
PCA bi-plot of rye genotypes based on PC1 and PC2 components and vectors of traits analyzed in the study. WFS classes for genotypes are indicated by color scheme shown below plot. Genotype numbers refer to Table S2.

In a PCA bi-plot analysis, the first two PCs determined 67.5% of the total variations for traits studied in the rye population (Figure 3). PC1 accounted for 48.1% of the total variation and was mainly associated with WFS, LTT, FLN, PGH, and DTA. PC2 with 19.4% share of total variation was mainly associated with DTA. The PHT and TIL vectors indicated associations with both PC1 and PC2. WFS, LTT, FLN, PGH, and DTA vectors were closely spaced and directed in the same orientation on the bi-plot (Figure 3), which suggested high association as confirmed by the correlation analysis (Table 3). A near right angle between DTA and PHT/TIL vectors in the bi-plot suggested a negative association (Figure 3) that was also supported by the correlation data (Table 3). However, the correlation test did not support a negative association between FLA and TIL as indicated by the bi-plot.

Winter genotypes were positioned in all four quadrants (Figure S8), with all of the very high WFS class positioned in the second and third quadrants (Figure 3). Genotypes with low or very low WFS class, including most spring lines, were positioned within the first and fourth quadrants. Perennial genotypes were clustered in the middle, and most facultative genotypes were positioned on the lower half of the plot (Figure S8). FLN, PGH, and DTA vectors were primarily associated with the winter-hardy genotypes in second quadrant, whereas PHT and TIL vectors were mainly directed towards a subgroup of the winter-hardy genotypes positioned in the third quadrant. This subgroup included genotypes from Northern Europe such as Anna, Pearl, and Ponsi, which are relatively tall (>133 cm; Table S2). In contrast, most of the winter-hardy Canadian genotypes primarily positioned in the second quadrant appeared to rely more on PGH and FLN traits for their high WFS (Figure 3).

### 2.9. Heritability Estimates Show Genotype Had High Influence on Traits Analyzed

All traits studied showed significant (*p* < 0.001) effects of genotype (G), environment (E), and genotype x environment (G × E) interactions as revealed by an ANOVA analysis (Table 4). The environmental factor was largest for WFS, due to the large variations in winter conditions during the trials (Figure S1). Estimations of broad-sense heritability (*h^2^*) generated values ranging from 0.45 to 0.84 for the different traits (Table 4). Heritability values above 0.6 are generally considered high and were estimated for DTA (0.84), FLN (0.81), FLA (0.76), PHT (0.74), and TIL (0.74). A medium range heritability (0.30–0.60) was estimated for WFS (0.48) and PGH (0.45).

## 3. Discussion

### 3.1. Rye Population Studied Provided a Wide Variation of Winter-Hardiness Levels

Cultivation of winter cereals at Northern latitudes is highly dependent on plant survival during winter, which can drastically affect seed or biomass yields at maturity. Thus, studies on WFS are important for future expansion of winter rye growing areas and production volumes, but also for development of other winter cereals with improved winter hardiness. Winter rye is unique among the temperate cereal crops as it has adapted well to Northern climates, allowing cultivation in areas not suitable for wheat. Thus, the most winter-hardy rye genotypes in the study hold valuable information regarding winter survival during very cold winters in the northern latitudes in Asia, Europe, and North America. In contrast, the tender genotypes in the population were useful for identification of traits not associated with high WFS. A large amount of the frost tolerance variation among cereals depends on copy number and allele differences at the *VRN1* and *FR-2* loci, respectively, based on studies of diploid and hexaploid wheat [50,51] and barley [52,53]. *Fr-R2* is confirmed as a major frost hardiness locus in rye [54,55], but very little is known about other loci affecting WFS or LTT in rye. In wheat, interactions between *VRN1* and *Fr-A2* alleles modulate cold-induced *CBF* gene expression that is critical for induction of COR genes and development of high LTT [20]. Thus, the poor WFS displayed by spring rye types in this study is likely due to the lack of vernalization requirement combined with the inability to induce *COR* gene expression to high levels when exposed to low temperatures in the autumn [56,57].

### 3.2. Efficiency of the Cold Acclimation Process Was a Major Factor for WFS

In the freezing tests, the LT_50_ values were determined on plants cold acclimated under constant exposure to 4 °C temperature, low light intensity, and a short-day cycle. These growth room conditions do not include the frequent variations in light intensity and temperature that naturally occur in the field, which constitute environmental signals that can prime plants to increase their cold acclimation [58]. Such triggering events may include a short cold spell prior to a longer period of cold or exposure to slightly subzero temperatures, initiating a second hardening step [30,59]. Despite the differences in cold acclimation conditions under natural and controlled conditions, the determined LTT correlated relatively well with WFS determined from individual trials (r = 0.54–0.82; *p* < 0.001) and particularly well to BLUEs determined for WFS (r = 0.90; *p* < 0.001; Table 2). Thus, the efficiency of the cold acclimation process in the autumn was the major contributing factor to WFS in this study. Previous field tests of winter wheat genotypes performed in the Saskatoon region also demonstrate a very high correlation (r = 0.95) between LTT and WFS [37]. Saskatoon winters typically have low humidity levels, thin snow cover, and overall unfavorable conditions for snow mold infections, which can have a large negative impact on WFS [32,60]. In locations with long-lasting snow cover and humid conditions at ground level, the correlations between WFS and LTT are expected to be considerably lower than observed in this study.

### 3.3. Developments at SAM Were Closely Associated with WFS and LTT

The rye genotypes Musketeer, AC Rifle, AC Remington with the highest FLN (≥12.0 leaves; Table S2) were among genotypes with highest WFS (WFS ≥83%) and highest LTT (lowest LT_50_ < −27.0 °C) (Table 1). A low FLN demonstrated by breeding lines Petkus and Carsten (8.0 and 8.6, respectively, Table S2) suggested a short cold acclimation process underlies low WFS and LTT for these genotypes. FLN in the rye population was strongly associated (*p* < 0.001) with WFS (r = 0.80) and LTT (r = 0.71), respectively (Table 3). Thus, the majority of the variation for WFS among rye genotypes related to differences in the length of the cold acclimation period is similar to observations made in other cereals [56,61]. The switch to inflorescence meristem identity at SAM coincides with an up-regulation of *VRN1* expression [9], and *VRN1* allele difference is one of the factors determining duration of the vegetative phase in hexaploid wheat [51]. The formation of leaf primordia at the peripheral flank of SAM occurs at auxin maxima see review [62], a process modulated by the relative levels of cytokinin and gibberellin, which display antagonistic effects at SAM [63]. The phytohormone methyl jasmonate (MeJA) is also proposed to affect floral transition time in wheat [64]. Thus, floral transition is strongly affected by phytohormone levels.

The shoot curvature at the base of the crown was not permanently induced by cold as the rye plants reverted back to erect growth habit when growth resumed at normal temperature and long-day conditions. An altered negative gravitropism response resulting in asymmetric distribution of auxin in the shoot was proposed to cause PGH based on studies of gravity persistent signal (*gps*) mutants in *Arabidopsis* [65]. Like FLN, PGH is also associated with *VRN1* or closely linked *Fr-1* locus on chromosome 5A according to early studies of winter wheat [66]. Later studies implicated sensitivity to photoperiod in addition to vernalization sensitivity as the two major factors controlling PGH during juvenile growth in wheat [67]. A role for vernalization requirement was indicated in the study, where winter and perennial types with vernalization requirements developed a stronger PGH than spring and facultative types (Table S2). A role for phytohormone involvement in PGH is supported by studies in barley, for which recessive alleles at the *sdw1*/*denso* locus associated with gibberellin biosynthesis induced early prostrate growth in addition to semi-dwarf growth and delayed flowering [68,69]. As phytohormone levels are strongly implicated in the determination of floral transition time and prostrate growth habit during cold acclimation [62,64,70], the influence of *VRN1* and/or *CBF* alleles on genes controlling phytohormone metabolism at SAM are likely to underlie some of the differences in FLN, PGH, and LTT observed for the rye population.

### 3.4. WFS and LTT Were Associated with PHT and TIL

The strong link between LTT and PHT (r = 0.59; *p* < 0.001), suggested PHT is affected by early events at SAM prior to floral transition. The intercalary meristems, from which leaf initials, axillary buds, and internodes are formed, are laid down during the early stages of SAM development [42], but stem elongation is paused until vernalization, temperature, and photoperiod requirements are fulfilled [71]. A signal transported from the shoot apex is suggested to control internode elongations starting with basal internodes elongating first and the peduncle last [42]. Gibberellin concentrations have a role in stem elongation by promoting increased cell division and cell elongations at the intercalary meristems of the stem [42,46]. The timing of stem elongation in the spring for winter type is important as early elongation can lead to frost kill due to exhausted LTT at this stage. Like FLN and PGH, a major locus for stem elongation in hexaploid wheat is located close to the *VRN-A1* locus on chromosome 5A [71]. PHT and TIL, which showed lower correlation with WFS than FLN and PGH (Table 3), appeared to be related to WFS for only a subgroup of the rye population (Figure 3).

### 3.5. DTA Showed Strongest Association with PGH Displayed during Cold Acclimation

The development of flowers in *Triticeae* species with fulfilled vernalization requirement starts when photoperiod and temperature requirements are met, which occurs in the spring for winter cereals [72]. At this transition, the up-regulated *VRN1* and absence of *VRN2* expression in leaves leads to induced expression of *VRN3* [73]. VRN3 is the cereal orthologue of *Arabidopsis* FLOWERING LOCUS T (FT), a transmissible florigen signal promoting flower development [74]. Besides variation for *VRN* alleles controlling vernalization requirement, allelic differences for *PHOTOPERIOD1* (*PPD1*), circadian clock-related *earliness* per se, early maturity, and gibberellin-regulated genes are some of the factors modifying flowering time in cereals (see review [75]). Like many developmental traits associated with WFS, gibberellin levels regulate development of inflorescence into flowers during long days [76]. Thus, the negative correlation between DTA and PHT (and TIL) observed for the rye population could be due to gibberellins stimulating both early flowering (low DTA) and stem elongation (high PHT/TIL). The strong association between DTA and PGH (r = 0,43; *p* < 0.001; Table 3) suggested genes affecting phytohormone levels at SAM during cold acclimation could also determine flowering time in the spring. Since the developmental traits induced during vernalization and associated with WFS are influenced by plant growth regulators [70], genes affecting phytohormone metabolism at SAM are of prime interest for future WFS studies.

## 4. Materials and Methods

### 4.1. Plant Material and Seed Production

A panel of 96 rye genotypes previously partially characterized for WFS [48] was used in the study (Table 1). The genotypes are represented by 72 winter, 8 spring, 8 facultative, and 8 perennial types and most of them originate from North America or Europe. The genotypes were propagated in growth rooms by seeding in 38-well trays (PL-38-STAR-DP; T.O. Plastics, Clearwater, MN, USA) containing LG3 Propagation Mix (Sungro Horticulture, Agawam, MA, USA) supplemented with 7.3 g L^−1^ slow-release fertilizer Type 100 NPK 14-14-14 (Arysta Lifescience America Inc., Burton, OH, USA) and 1.0 g L^−1^ Micromax micronutrients (ICL Specialty Fertilizers, Dublin, OH, USA). Plant growth to the four-leaf stage was performed under 20/18 °C day/night temperatures, 16/8 h day/night cycle, 50% relative humidity, and 250 μEm^−2^ s^−1^ light irradiance. Thereafter, growth was continued at 4 °C, 8/16 h day/night cycle, 120 μE m^−2^ s^−1^ light intensity, and 50% humidity to allow for seven weeks’ cold acclimation. Acclimated plants were then transplanted into six-inch pots containing soil and slow-release fertilizers as described above and grown to maturity in a greenhouse set at 17/7 h light/dark regime, 1500 μE m^−2^ s^−1^ light irradiance, 23/18 °C day/night temperatures, and 50% relative humidity. During the active growth, liquid fertilizer (0.36 g L^−1^ NPK 20-20-20) was applied once per week. Pollination control was performed by placing five plants per genotype within a 1.2 m^3^ pollination bag just prior to anthesis as described [77]. Seeds harvested from mature spikes were stored at room temperature until use.

### 4.2. Field Trials for Determination of WFS

Field trials of 96 rye genotypes (Table 1) were conducted at the University of Saskatchewan Experimental Farm, Saskatoon, Saskatchewan, Canada (52°10′ N, 106°30′ W, 457 m altitude) as previously described [48]. Climatological data were collected during the trials (Table S1).

### 4.3. Freezing Tests for Determination of LTT

To determine the LTT (negative LT_50_ values), freezing tests were performed on the 96 rye genotypes. Cold-hardy winter wheat cv. Norstar was used as internal control. In this assay, plants were grown to four-leaf stage in a growth chamber and cold acclimated for five weeks at 4 °C as described above. A total of 60 cold-acclimated plants per genotype were then transplanted into five six-inch pots with 12 plants per pot and acclimatized in the dark to −3 °C for 12 h in an EPZ-4H test chamber (ESPEC North America Inc., Hudsonville, MI, USA). Thereafter, the chamber temperature was reduced at a rate of 2.0 °C h^−1^. When the first test temperature was reached, one pot per genotype was removed from the freezing chamber and transferred to a 4 °C growth chamber for thawing. The remaining pots in the freezing chamber were removed one by one upon every 1.5 °C additional decrease in temperature. For genotypes with highest LTT, the five freezing test temperatures ranged from −24 to −30 °C, whereas test temperatures ranged from −13.5 to −19.5 °C for genotypes with the lowest LTT. The selected test temperatures were predetermined based on WFS data and small-scale freezing tests.

Frost-exposed plants were maintained at 4 °C, 8/16 h day/night cycle, 120 μE m^−2^ s^−1^ light intensity, and 50% humidity for 20 h before being trimmed to one-inch height and transferred to a growth room set at 20/18 °C day/night temperature, 16/8 h day/night cycle, 50% humidity, and 250 μEm^−2^ s^−1^ light irradiance. The plants were fertilized once per week with NPK 20-20-20 (35 g L^−1^) during watering. After two weeks of regrowth, plant recovery was rated for each of the 60 plants per genotype using a scale from zero to five, where zero indicated no regrowth and five represented full regrowth from all tillers. Average survival scores obtained at each test temperature were plotted against freezing test temperature to generate a kill curve. The freezing temperature at which 50% of the plants survived was determined as the LT_50_ value. The freezing tests were performed twice to determine an average LT_50_ value for each genotype.

### 4.4. Collection of Phenotypic Data for Developmental Traits

The developmental traits FLN, PGH, PHT, TIL, and DTA were assessed on plants cold acclimated in a growth room and grown to maturity in a greenhouse. Each trait was examined in four separate trials with five plants per genotype in each test. To determine FLN, plant leaves were labeled numerically as they developed from the primary stem and the number of the final flag leaf on the main stem was recorded as FLN. PGH was rated for fully cold-acclimated plants by visual scoring of three different growth habits: (1) erect, (2) intermediate, or (3) prostrate. The number of days from the end of cold acclimation and first anther extrusion was recorded as DTA. At maturity, the length of the three longest stems per plant were measured from the soil surface to the top of the head with awn length excluded. The average PHT and TIL were recorded for the genotype. Flag leaf area (FLA) was determined on field-grown plants upon full extension of inflorescence and performed on three plants per genotype with five flag leaves sampled per plant. A LI-3000A Portable Area Meter connected to LI-3050A Transparent Belt Conveyer instrument (LI-COR Inc., Lincoln, NE, USA) was used for the FLA measurements.

### 4.5. Statistical Analyses

The phenotypic data collected were tested for normality using the Minitab 19 Statistical Software (Minitab, LLC, State College, PA, USA). Analysis of variance (ANOVA) was performed by the general linear model using the GEA-R software (Genotype x Environment Analysis with R for Windows) version 4.1 (CIMMYT Research Data & Software Repository Network El Batan, Mexico). Mean sum of squares from ANOVA were applied to calculate heritability (*h*^2^) for each trait [78]. To determine the overall trait score for each genotype, the Best Linear Unbiased Estimates (BLUEs) were calculated for all the phenotype data collected [79]. The calculation was conducted using the statistical analysis software META-R (Multi Environment Trial Analysis with R) version 6.04 (CIMMYT Research Data & Software Repository Network, El Batan, Mexico). Correlation analyses and principal component analysis (PCA) were conducted using RStudio package version 3.5.1 software [80]. The first two components were used to create bi-plot illustrating the relationships between genotypes and measured traits.

## 5. Conclusions

This study of WFS over five years combined with studies of LTT and six developmental traits showed WFS was almost entirely determined by the cold acclimation process. Thus, variation in WFS among the rye genotypes could to a large extent be explained by differences in the number of leaf initials produced at SAM during cold acclimation, factors inducing PGH in the shoot, and possibly also factors controlling early internode development at SAM. FLA and DTA are traits developed in the spring in winter types and showed no or low association with WFS, but variation for the traits is expected to impact agronomic performance and grain yield. The factors underlying the highly correlated traits FLN, PGH, and maybe also PHT are important to understand development of LTT in winter cereals. A moderate to high heritability displayed by the developmental traits indicated genetics had a large influence on trait values. This also suggests that the highly heritable developmental traits can be used in the selection of lines with high WFS among autumn-seeded rye. With high heritability estimates, good segregation for trait values within the population, and available genome sequence assembly [81], the requirements needed for a genome-wide association study are met [82]. Further genetic studies are expected to reveal the genetic basis for WFS and associated developmental traits for future utilization to enhance WFS in other winter cereals and expand their cultivation in temperate regions.

## Figures and Tables

**Table 2:** Correlations between LTT and WFS.

Trait	WFS 2014/15	WFS 2015/16	WFS 2016/17	WFS 2017/18	WFS 2018/19	WFS-BLUE
Low temperature tolerance (LTT) ^1^	0.70 ***	0.62 ***	0.82 ***	0.54 ***	0.81 ***	0.90 ***
Winter field survival 2014/15		0.44 **	0.54 ***	0.37 **	0.63 ***	0.66 ***
Winter field survival 2015/16			0.66 ***	0.17	0.49 ***	0.76 ***
Winter field survival 2016/17				0.36 **	0.67 ***	0.91 ***
Winter field survival 2017/18					0.58 ***	0.59 ***
Winter field survival 2018/19						0.88 ***
Winter field survival BLUE score ^2^						

^1^ LTT is defined as negative LT_50_ value; ^2^ BLUE score determined from five WFS trials. ** significance of Pearson correlation coefficient *p* < 0.01; *** *p* < 0.001.

**Table 3:** Correlations between WFS, LTT, and developmental traits.

	LTT	FLN	PGH	DTA	PHT	TIL	FLA
Winter field survival (WFS) ^1^	0.90 ***	0.80 ***	0.61 ***	0.25 *	0.34 ***	0.30 **	0.13
Low temperature tolerance (LTT) ^2^		0.71 ***	0.59 ***	0.17	0.39 ***	0.36 ***	0.14
Final leaf number (FLN) ^1^			0.43 ***	0.14	0.28 **	0.26 *	0.11
Prostrate growth habit (PGH) ^1^				0.43 ***	0.03	0.06	0.11
Days to anthesis (DTA) ^1^					−0.29 **	−0.25 *	0.13
Plant height (PHT) ^1^						0.72 ***	0.05
Top internode length (TIL) ^1^							0.05
Flag leaf area (FLA) ^1^							

^1^ BLUE scores. ^2^ Negative LT_50_ values. Pearson correlation coefficient were determined at * *p* < 0.05; ** *p* <0.01; *** *p* < 0.001.

**Table 4:** Analysis of variance and heritability of WFS and developmental traits.

Trait ^1^	Mean Sum of Squares			
	Genotype (G)	Environment (E)	G × E	Heritability (*h*^2^)
Winter field survival (WFS)	65,990.78 ***	3,303,871.00 ***	13,242.97 ***	0.48
Final leaf number (FLN)	177.85 ***	196.48 ***	9.30 ***	0.81
Prostrate growth habit (PGH)	28.02 ***	15.57 ***	6.38 ***	0.45
Days to anthesis (DTA)	6546.93 ***	2173.40 ***	285.18 ***	0.84
Plant height (PHT)	23,717.40 ***	15,098.94 ***	1815.25 ***	0.74
Top internode length (TIL)	4077.32 ***	871.38 ***	314.83 ***	0.74
Flag leaf area (FLA)	2547.20 ***	1901.13 ***	184.37 ***	0.76

^1^ BLUE scores. Pearson correlation coefficient determined at *** *p* < 0.001.

## Data Availability

All data is included in the manuscript text and Supplementary Tables.

## Supplementary Materials

The following are available online at www.mdpi.com/article/10.3390/plants10112455/s1. Figure S1: Frequency distribution for WFS data obtained for rye population in five field trials. Correlation analysis is presented in Table 2; Figure S2: Frequency distribution and correlation analysis for FLN data obtained for rye population in four studies; Figure S3: Frequency distribution and correlation analysis for PGH data obtained for rye population in four studies. Phenotypes scored for PGH is shown; Figure S4: Frequency distribution and correlation analysis for days to DTA data obtained for rye population in four studies; Figure S5: Frequency distribution and correlation analysis for PHT data obtained for rye population in four studies; Figure S6: Frequency distribution and correlation analysis TIL data obtained for rye population in four studies; Figure S7: Frequency distribution and correlation analysis for FLA data obtained for rye population in four field trials; Figure S8: PCA bi-plot of rye genotypes based on PCA1 and PCA2 components and vectors of traits analyzed in the study. Genotypes with winter (W), spring (S), facultative (F), and perennial (P) growth habit are indicated; Table S1: Local climatological data during consecutive growing seasons 2014 to 2019; Table S2: Development trait data for different growth habit classes of rye.
