# Supplementary material for: The Relationships between Plant Developmental Traits and Winter Field Survival in Rye (Secale cereale L.)
Source: Plants (Basel). 2021 Nov 13;10(11):2455. doi: 10.3390/plants10112455 (PMC8625450; doi:10.3390/plants10112455)
Supplement: Supplementary file 1 [file plants-10-02455-s001.zip › Figure S2.pdf]

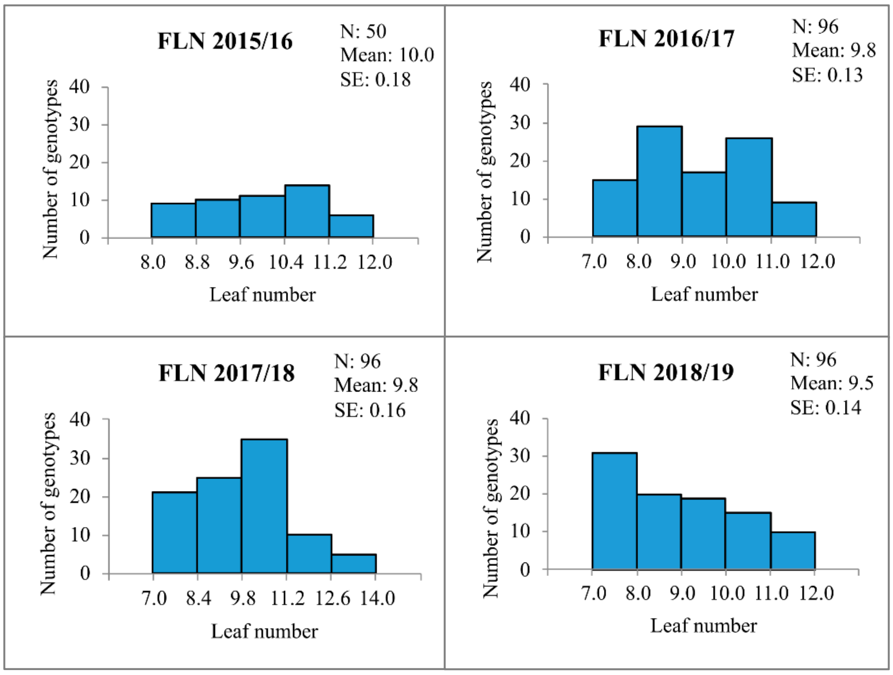

Correlations between FLN values determined in different trials

Trait_trial	FLN_15/16	FLN_16/17	FLN_17/18	FLN_18/19	FLN_BLUE
FLN_15/16	1.00				
FLN_16/17	0.82^{***}	1.00			
FLN_17/18	0.71^{***}	0.85^{***}	1.00		
FLN_18/19	0.67^{***}	0.79^{***}	0.88^{***}	1.00	
FLN_BLUE	0.86^{***}	0.91^{***}	0.95^{***}	0.93^{***}	1.00

Pearson correlation coefficient determined at *** $p < 0.001$.

Figure S2. Frequency distribution and correlation analysis for FLN data obtained for rye population in four studies.
