# Supplementary material for: The Relationships between Plant Developmental Traits and Winter Field Survival in Rye (Secale cereale L.)
Source: Plants (Basel). 2021 Nov 13;10(11):2455. doi: 10.3390/plants10112455 (PMC8625450; doi:10.3390/plants10112455)
Supplement: Supplementary file 1 [file plants-10-02455-s001.zip › Figure S3.pdf]

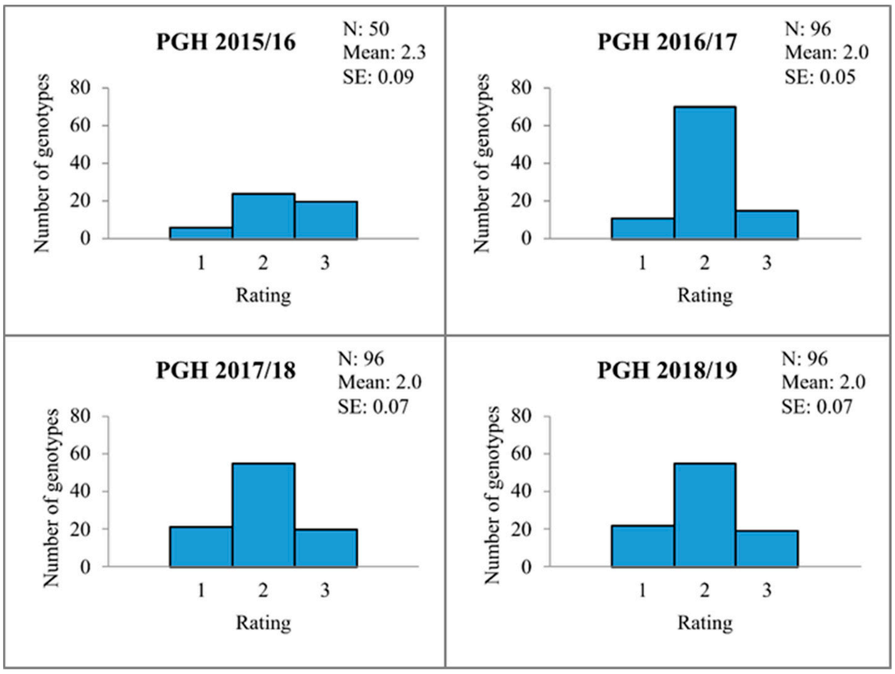

Correlations between PGH values determined in different trials.					
Trait_trial	PGH_15/16	PGH_16/17	PGH_17/18	PGH_18/19	PGH_BLUE
PGH_15/16	1.00				
PGH_16/17	0.56***	1.00			
PGH_17/18	0.15	0.22*	1.00		
PGH_18/19	0.50***	0.43***	0.68***	1.00	
PGH_BLUE	0.74***	0.67***	0.76***	0.89***	1.00

Pearson correlation coefficient determined at * $p<0.05$, *** $p < 0.001$.

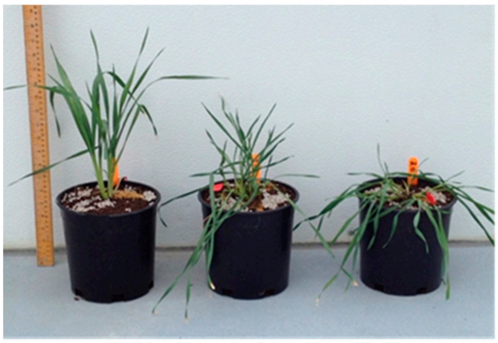

Figure S3. Frequency distribution and correlation analysis for PGH data obtained for rye population in four studies. Phenotypes scored for PGH is shown.
