# Supplementary material for: The Relationships between Plant Developmental Traits and Winter Field Survival in Rye (Secale cereale L.)
Source: Plants (Basel). 2021 Nov 13;10(11):2455. doi: 10.3390/plants10112455 (PMC8625450; doi:10.3390/plants10112455)
Supplement: Supplementary file 1 [file plants-10-02455-s001.zip › Figure S4.pdf]

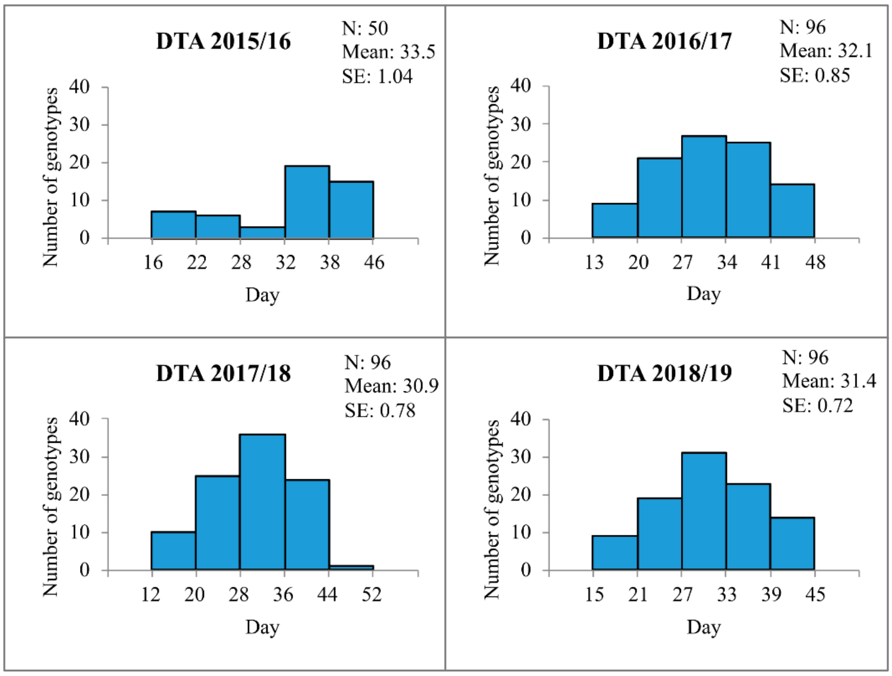

Correlations between DTA values determined in different trials.

Trait_trial	DTA_15/16	DTA_16/17	DTA_17/18	DTA_18/19	DTA_BLUE
DTA_15/16	1.00				
DTA_16/17	0.92^{***}	1.00			
DTA_17/18	0.70^{***}	0.77^{***}	1.00		
DTA_18/19	0.95^{***}	0.92^{***}	0.85^{***}	1.00	
DTA_BLUE	0.95^{***}	0.89^{***}	0.92^{***}	0.96^{***}	1.00

Pearson correlation coefficient determined at *** $p < 0.001$.

Figure S4. Frequency distribution and correlation analysis for days to DTA data obtained for rye population in four studies.
