# Supplementary material for: The Relationships between Plant Developmental Traits and Winter Field Survival in Rye (Secale cereale L.)
Source: Plants (Basel). 2021 Nov 13;10(11):2455. doi: 10.3390/plants10112455 (PMC8625450; doi:10.3390/plants10112455)
Supplement: Supplementary file 1 [file plants-10-02455-s001.zip › Figure S5.pdf]

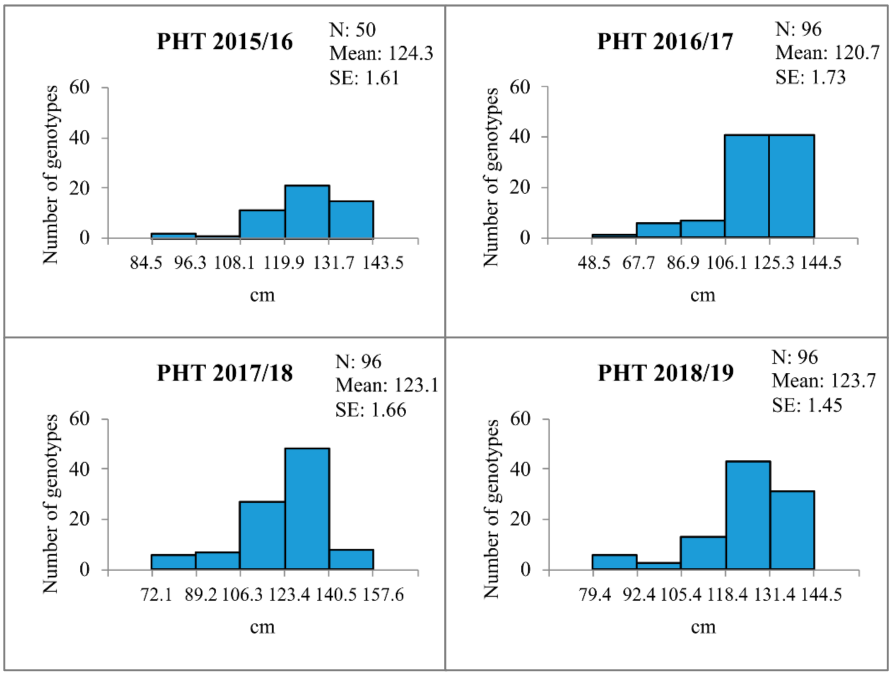

Correlations between PHT values determined in different trials.

Trait_trial	PHT_15/16	PHT_16/17	PHT_17/18	PHT_18/19	PHT_BLUE
PHT_15/16	1.00				
PHT_16/17	0.74^{***}	1.00			
PHT_17/18	0.18	0.68^{***}	1.00		
PHT_18/19	0.59^{***}	0.81^{***}	0.82^{***}	1.00	
PHT_BLUE	0.80^{***}	0.91^{***}	0.88^{***}	0.94^{***}	1.00

Pearson correlation coefficient determined at *** $p < 0.001$.

Figure S5. Frequency distribution and correlation analysis for PHT data obtained for rye population in four studies.
