# Supplementary material for: The Relationships between Plant Developmental Traits and Winter Field Survival in Rye (Secale cereale L.)
Source: Plants (Basel). 2021 Nov 13;10(11):2455. doi: 10.3390/plants10112455 (PMC8625450; doi:10.3390/plants10112455)
Supplement: Supplementary file 1 [file plants-10-02455-s001.zip › Figure S6.pdf]

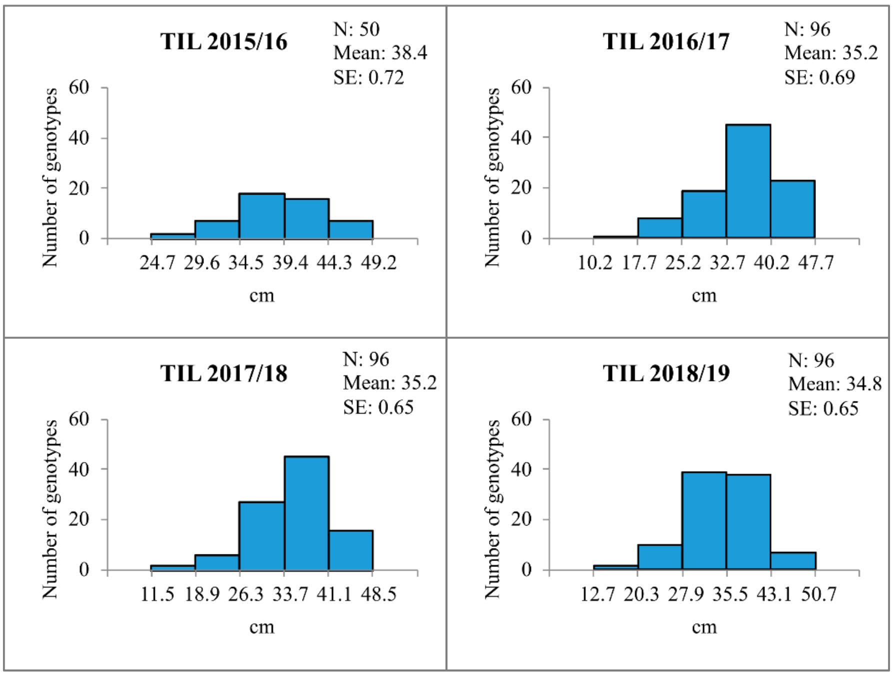

Correlations between TIL values determined in different trials.

Trait_trial	TIL_15/16	TIL_16/17	TIL_17/18	TIL_18/19	TIL_BLUE
TIL_15/16	1.00				
TIL_16/17	0.54^{***}	1.00			
TIL_17/18	0.24	0.77^{***}	1.00		
TIL_18/19	0.58^{***}	0.78^{***}	0.78^{***}	1.00	
TIL_BLUE	0.89^{***}	0.97^{***}	0.77^{***}	0.79^{***}	1.00

Pearson correlation coefficient determined at $^{***}p < 0.001$.

Figure S6. Frequency distribution and correlation analysis TIL data obtained for rye population in four studies.
