# Supplementary material for: The Relationships between Plant Developmental Traits and Winter Field Survival in Rye (Secale cereale L.)
Source: Plants (Basel). 2021 Nov 13;10(11):2455. doi: 10.3390/plants10112455 (PMC8625450; doi:10.3390/plants10112455)
Supplement: Supplementary file 1 [file plants-10-02455-s001.zip › Figure S7.pdf]

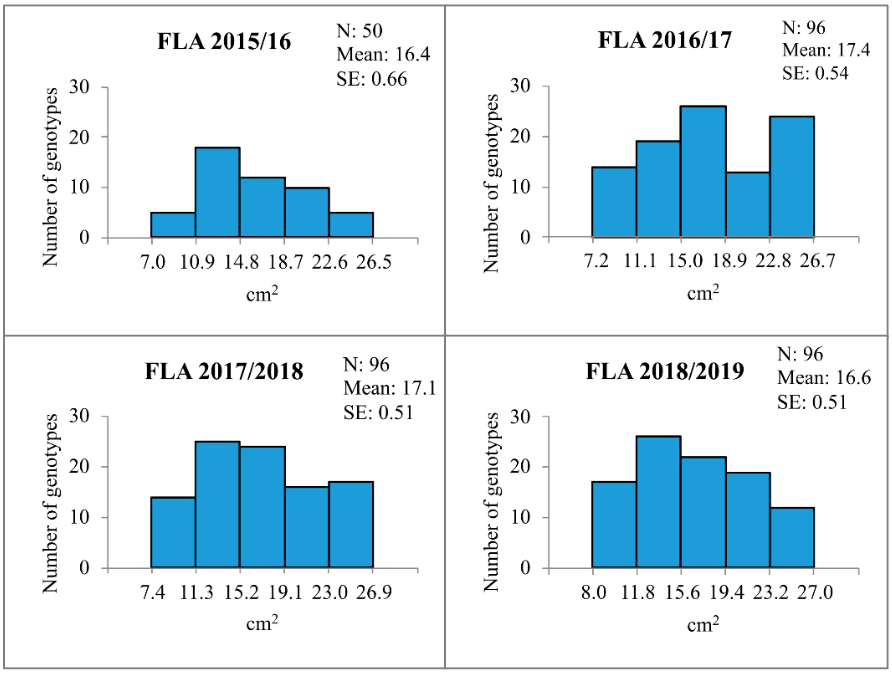

Correlations between FLA values determined in different trials.

Trait_trial	FLA_15/16	FLA_16/17	FLA_17/18	FLA_18/19	FLA_BLUE
FLA_15/16	1.00				
FLA_16/17	0.61^{***}	1.00			
FLA_17/18	0.45^{**}	0.90^{***}	1.00		
FLA_18/19	0.60^{***}	0.88^{***}	0.88^{***}	1.00	
FLA BLUE	0.62^{***}	0.94^{***}	0.93^{***}	0.94^{***}	1.00

Pearson correlation coefficient determined at $^{**}p < 0.01$; $^{***}p < 0.001$.

Figure S7. Frequency distribution and correlation analysis for FLA data obtained for rye population in four field trials.
