# Supplementary material for: The Relationships between Plant Developmental Traits and Winter Field Survival in Rye (Secale cereale L.)
Source: Plants (Basel). 2021 Nov 13;10(11):2455. doi: 10.3390/plants10112455 (PMC8625450; doi:10.3390/plants10112455)
Supplement: Supplementary file 1 [file plants-10-02455-s001.zip › Figure S8.pdf]

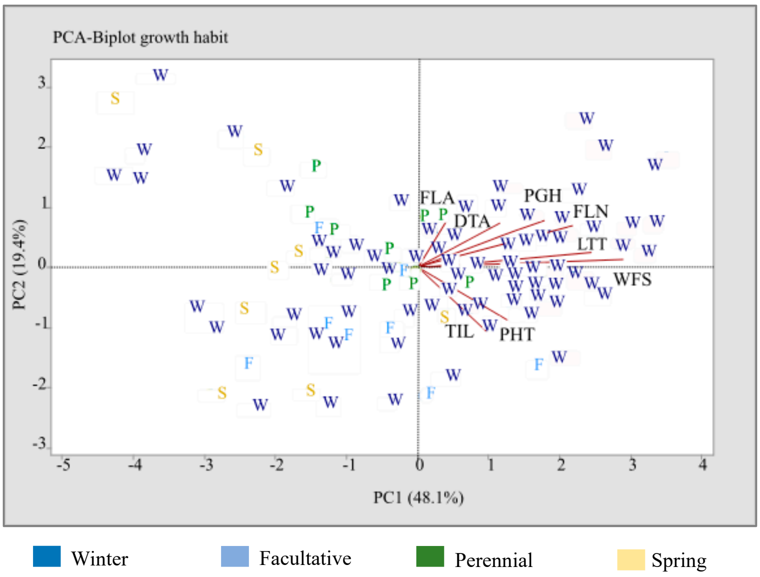

Figure S8. PCA bi-plot of rye genotypes based on PCA1 and PCA2 components and vectors of traits analyzed in the study. Genotypes with winter (W), spring (S), facultative (F), and perennial (P) growth habit are indicated.
