# Supplementary material for: The Relationships between Plant Developmental Traits and Winter Field Survival in Rye (Secale cereale L.)
Source: Plants (Basel). 2021 Nov 13;10(11):2455. doi: 10.3390/plants10112455 (PMC8625450; doi:10.3390/plants10112455)
Supplement: Supplementary file 1 [file plants-10-02455-s001.zip › Table S1.pdf]

Table S1. Local climatological data during consecutive growing seasons 2014 to 2019*.

	Sep	Oct	Nov	Dec	Jan	Feb	Mar	Apr	May	Jun	Jul	Aug
**2014/2015**												
Maximum temperature (°C)	30	24	8	7	6	1	16	27	28	32	33	32
Minimum temperature (°C)	-2	-4	-29	-32	-34	-30	-30	-9	-4	4	5	2
Average maximum temperature (°C)	19	13	-6	-6	-7	-11	2	12	18	24	25	24
Average minimum temperature (°C)	6	1	-13	-13	-16	-22	-6	-1	2	11	13	11
Maximum soil temperature (°C)	16	11	3	-1	-1	-8	3	9	17	21	23	20
Minimum soil temperature (°C)	8	2	-10	-13	-19	-15	-12	0	6	12	17	12
Average soil temperature (°C)	12	8	-3	-4	-7	-10	0	5	11	18	20	17
Precipitation (mm)	13.0	15.2	14.2	0.0	1.0	0.3	1.0	19.6	8.9	33.5	37.1	68.6
**2015/2016**												
Maximum temperature (°C)	27	26	10	6	4	5	11	25	33	30	29	29
Minimum temperature (°C)	-5	-5	-24	-27	-31	-24	-17	-7	-2	5	8	3
Average maximum temperature (°C)	18	13	1	-5	-8	-3	2	12	22	24	24	23
Average minimum temperature (°C)	6	1	-6	-13	-12	-12	-6	-1	6	11	12	11
Maximum soil temperature (°C)	16	11	7	3	-2	-2	2	8	16	22	22	20
Minimum soil temperature (°C)	6	3	-2	-6	-7	-7	-6	0	5	15	17	15
Average soil temperature (°C)	11	6	2	-3	-4	-4	0	4	12	18	19	18
Precipitation (mm)	45.5	32.0	11.7	0.3	1.0	2.0	2.3	2.0	39.4	50.0	55.4	51.6
**2016/2017**												
Maximum temperature (°C)	29	17	20	2	6	8	12	21	30	33	30	27
Minimum temperature (°C)	-2	-3	-10	-30	-35	-31	-26	-9	-3	4	6	6
Average maximum temperature (°C)	19	6	7	-9	-7	-5	-1	10	19	22	26	25
Average minimum temperature (°C)	5	-1	-2	-18	-17	-13	-10	-2	5	10	12	10
Maximum soil temperature (°C)	18	12	6	-1	-3	-1	3	6	18	22	23	20
Minimum soil temperature (°C)	9	2	-1	-12	-7	-6	-6	0	6	11	18	15
Average soil temperature (°C)	12	2	1	-7	-5	-3	-2	2	10	15	21	18
Precipitation (mm)	26.4	30.7	8.6	0.0	1.8	0.0	1.0	15.7	55.9	45.7	24.6	14.7
**2018/2019**												
Maximum temperature (°C)	23	21	4	5	4	-11	14	22	31	34	29	34
Minimum temperature (°C)	-9	-10	-23	-30	-31	-39	-34	-10	-10	4	6	3
Average maximum temperature (°C)	12	9	-4	-5	-8	-22	7	12	17	22	24	24
Average minimum temperature (°C)	3	-4	-10	-14	-19	-31	-17	-3	1	9	12	10
Maximum soil temperature (°C)	15	5	3	-2	-3	_	_	10	18	19	22	_
Minimum soil temperature (°C)	3	1	-2	-6	-9	_	_	0	4	12	15	_
Average soil temperature (°C)	9	4	-1	-3	-7	_	_	4	11	15	18	_
Precipitation (mm)	31.0	6.4	4.8	0.3	2.3	0.0	4.6	2.3	7.6	63.2	67.8	3.8

*Data from www.weatherlink.com.
