# Supplementary material for: The Relationships between Plant Developmental Traits and Winter Field Survival in Rye (Secale cereale L.)
Source: Plants (Basel). 2021 Nov 13;10(11):2455. doi: 10.3390/plants10112455 (PMC8625450; doi:10.3390/plants10112455)
Supplement: Supplementary file 1 [file plants-10-02455-s001.zip › Table S2.pdf]

Table S2. Development trait data for different growth habit classes of rye.

Genotype[1/]	Growth habit	WFS class	FLN Leaves	PGH Score	DTA Days	PHT cm	TIL cm	FLA cm^2
1-Leth Coulee Rye	Winter	1	11.9	1.4	32.3	135.6	34.6	24.1
2-Gauthier	Winter	1	11.4	2.4	31.5	116.6	32.5	8.2
3-AC Remington	Winter	1	12.0	3.0	35.0	106.5	35.9	19.9
4-AC Rifle	Winter	1	12.3	2.8	36.7	113.4	33.5	12.6
5-Musketeer	Winter	1	12.4	2.5	35.1	124.7	37.8	22.3
6-SM 38R	Winter	1	11.9	2.1	30.7	126.6	35.4	24.1
7-Prima	Winter	1	11.8	2.5	36.2	128.8	36.5	18.5
8-Saratovskaja 4	Winter	1	10.5	2.8	33.0	129.9	41.5	22.9
9-SM 4R	Winter	1	10.7	2.5	36.9	122.5	36.0	16.4
10-Pearl	Winter	1	10.8	2.5	31.4	133.5	42.8	25.4
11-Kustro	Winter	1	10.3	2.5	38.6	130.1	37.1	15.9
12-Kharkivska 95	Winter	1	11.2	2.5	39.6	104.4	35.1	26.3
13-Kharkivska 98	Winter	1	11.5	2.5	33.0	120.5	40.0	21.3
14-Esprit	Winter	1	10.6	2.1	32.7	97.3	23.5	8.3
15-Ponsi	Winter	1	11.7	2.0	33.9	135.0	45.1	20.5
16-Hazlet	Winter	1	10.5	2.8	36.8	113.8	34.5	16.5
17-Antelope	Winter	1	9.2	2.8	31.6	124.7	44.4	15.6
18-Emerald	Winter	1	10.8	2.1	30.2	125.0	36.5	16.8
19-Anna	Winter	1	11.8	2.0	24.4	137.3	42.4	16.3
20-R003-4	Winter	2	9.8	2.0	31.2	123.3	38.5	10.2
21-Voima	Winter	2	10.1	2.3	41.2	137.8	34.3	20.9
22-Dakota	Winter	2	11.3	2.0	33.7	126.1	34.0	8.1
23-Sc-73	Winter	2	10.8	2.7	27.2	124.0	35.8	10.9
24-Animo	Winter	2	8.0	3.0	40.0	121.0	34.9	21.5
25-Caribou	Winter	2	10.9	3.0	41.1	125.5	44.7	9.9
26-Puma	Winter	2	10.8	2.8	27.7	134.3	37.0	20.0
27-Othello	Winter	2	11.2	2.3	36.4	127.7	42.2	16.4
28-Rymin	Winter	2	9.7	3.1	33.7	118.9	32.4	10.9
29-Adams	Winter	2	11.2	2.1	22.2	124.5	34.8	8.6
30-Sangaste	Winter	2	10.8	2.1	25.8	136.4	45.5	20.4
31-Visa	Winter	2	9.6	2.8	36.0	132.2	41.3	15.7
32-Vitallo	Winter	2	11.3	1.8	35.0	131.4	41.4	23.4
33-Halo	Winter	2	10.7	2.0	20.8	122.1	28.9	21.4
35-Frontier	Winter	2	8.8	1.8	32.2	126.8	43.4	21.0
36-Enzi	Winter	2	10.6	2.1	33.8	133.9	35.8	16.1
38-Motto	Winter	2	9.8	2.3	38.8	122.9	37.4	15.6
39-Dankowskie Selekcyjne	Winter	2	10.7	2.3	30.7	125.2	45.4	13.7
40-Galma	Winter	3	7.8	2.1	33.8	113.0	36.5	10.0
41-Cougar	Winter	3	10.6	2.3	20.1	119.9	34.9	16.1
42-Dominant	Winter	3	10.1	2.0	27.7	131.2	41.0	10.4
43-Dankowskie Nowe	Winter	3	11.2	2.3	39.0	134.1	38.5	21.1
44-Danko	Winter	3	9.8	2.1	31.5	113.7	38.8	18.6
46-Dankowskie Srebrne	Winter	3	10.0	2.3	31.9	135.9	43.0	24.2
47-Carolkurz	Winter	3	10.1	2.5	33.3	119.6	33.0	21.1
48-Horton	Winter	3	9.9	2.3	26.7	141.6	38.5	16.7
49-Kodiak	Winter	3	11.8	1.8	32.6	133.0	34.4	15.2
50-GC-100	Winter	3	8.4	2.0	43.1	127.3	36.1	24.7
51-Amilo	Winter	3	8.4	2.1	35.7	116.6	37.3	22.6
52-Sellino	Winter	3	8.7	2.0	31.3	123.1	34.5	18.0
54-Protector	Winter	3	8.2	2.3	32.9	139.1	39.4	8.0
55-Toivo	Winter	3	9.1	1.5	22.2	141.5	36.8	18.5

Table S2 cont.

56-Culpan	Winter	3	9.6	2.4	24.3	115.0	27.5	25.3
57-Hardy white spring Rye	Winter	3	9.4	2.1	35.3	119.0	32.8	19.0
59-Stoir	Winter	4	8.7	2.3	32.9	131.9	30.0	14.8
60-Vaschod	Winter	4	9.3	2.1	33.8	119.6	36.0	22.6
64-Carsten	Winter	4	8.6	2.0	33.7	134.1	37.4	24.4
67-Danae	Winter	4	8.0	1.4	25.0	118.0	29.6	11.5
68-Clse 35	Winter	4	9.4	1.1	21.2	129.6	42.4	11.5
71-L-286-R	Winter	4	8.8	2.1	41.8	85.4	19.1	14.1
73-Syn 20-L	Winter	4	8.3	2.4	34.0	87.6	23.9	13.0
75-Dakold	Winter	4	8.4	1.3	24.8	123.5	38.8	13.4
76-Wheeler	Winter	4	9.2	2.1	33.3	119.7	36.7	12.0
77-M.Karlic CT2	Winter	4	7.9	1.4	48.8	78.3	30.0	21.7
79-Petkus Kurzstroh	Winter	5	7.7	1.4	19.8	118.8	44.8	15.9
81-Petkus	Winter	5	8.0	1.3	25.3	121.2	42.9	13.2
85-Somro	Winter	5	8.3	2.1	21.7	111.8	31.7	15.7
87-Baltia	Winter	5	7.8	1.4	21.2	130.9	46.5	9.0
90-Ottawa Select	Winter	5	7.9	1.4	24.8	121.1	37.5	18.8
91-Gulzow Kunz CT1	Winter	5	8.7	1.4	31.2	67.2	10.5	15.2
94-L-145-N	Winter	5	8.2	1.7	42.8	91.7	20.7	15.7
95-L-145-P	Winter	5	8.5	1.7	35	94.6	22.8	14.4
96-L-18-R	Winter	5	7.7	2.1	35	81.5	19.8	14.9
		Average:	9.9	2.1	32.1	121.0	35.8	16.9
		Min:	7.7	1.1	19.8	67.2	10.5	8.0
		Max:	12.4	3.1	48.8	141.6	46.5	26.3
34-Balbo	Facultative	2	10.0	2.0	26.4	140.5	43.9	20.6
37-Explorer	Facultative	2	9.7	1.7	29.0	130.5	38.7	9.0
58-Maton	Facultative	3	8.2	1.3	41.2	120.3	25.0	14.2
63-Oklon	Facultative	4	8.2	2.3	23.7	134.0	36.5	15.0
69-Gator	Facultative	4	8.4	1.8	17.2	125.4	38.4	15.4
70-Elbon	Facultative	4	8.7	2.1	27.8	105.0	24.8	23.5
78-Wintergrazer 70	Facultative	5	8.2	1.4	26.2	144.4	34.8	10.7
83-Wren Abruzzi	Facultative	5	8.0	1.3	22.3	130.2	37.4	15.6
		Average:	8.7	1.7	26.7	128.8	34.9	15.5
		Min:	8.0	1.3	17.2	105.0	24.8	9.0
		Max:	10.0	2.3	41.2	144.4	43.9	23.5
66-Harach	Spring	4	8.6	2.1	25.7	120.9	42.7	16.3
74-SR4A-S5	Spring	4	8.0	2.1	33.2	86.3	25.4	20.2
80-Gazelle	Spring	5	8.8	1.7	25.0	120.6	42.7	17.4
82-Prolfic Spring	Spring	5	8.3	1.8	24.4	128.9	40.4	17.4
84-Extra Early Rye1	Spring	5	8.3	1.1	15.3	114.6	32.8	15.5
89-Fl-Synt	Spring	5	8.5	1.7	21.7	130.6	33.7	24.3
92-Rogo	Spring	5	8.8	1.1	20.5	122.6	35.5	22.1
93-Florida 401	Spring	5	8.0	1.0	16.1	130.3	39.3	13.1
		Average:	8.4	1.6	22.7	119.4	36.6	18.3
		Min:	8.0	1.0	15.3	86.3	25.4	13.1
		Max:	8.8	2.1	33.2	130.6	42.7	24.3
45-ACE-1	Perennial	3	11.4	1.7	40.0	129.5	36.5	13.2
53-R538	Perennial	3	8.3	2.4	36.7	113.4	35.5	25.7
61-R550	Perennial	4	8.0	2.7	34.8	123.4	26.5	14.5
62-Reimann Philipp	Perennial	4	9.6	2.1	34.2	113.9	30.4	14.2
65-R903	Perennial	4	8.3	2.1	40.2	120.6	27.6	22.4

Table S2 cont.

72-R904	Perennial	4	8.0	2.1	34.8	127.4	28.8	15.5
86-R1210	Perennial	5	8.5	2.0	43.3	113.4	35.5	10.0
88-R797	Perennial	5	8.3	2.1	43.7	115.2	35.7	15.9
		Average:	8.8	2.1	38.5	119.6	32.1	16.4
		Min:	8.0	1.7	34.2	113.4	26.5	10.0
		Max:	11.4	2.7	43.7	129.5	36.5	25.7
	Overall average:		9.6	2.1	31.4	121.4	35.5	16.9
	Overall min:		7.7	1.0	15.3	67.2	10.5	8.0
	Overall max:		12.4	3.1	48.8	144.4	46.5	26.3

[1/] Genotype number refers to number in PCA bi-plot (Fig. 3).
